# Differentiation of multipotent neural stem cells derived from Rett syndrome patients is biased toward the astrocytic lineage

**DOI:** 10.1186/s13041-015-0121-2

**Published:** 2015-05-27

**Authors:** Tomoko Andoh-Noda, Wado Akamatsu, Kunio Miyake, Takuya Matsumoto, Ryo Yamaguchi, Tsukasa Sanosaka, Yohei Okada, Tetsuro Kobayashi, Manabu Ohyama, Kinichi Nakashima, Hiroshi Kurosawa, Takeo Kubota, Hideyuki Okano

**Affiliations:** Division of Medicine and Engineering Science, Interdisciplinary Graduate School of Medicine and Engineering, University of Yamanashi, 4-4-37 Takeda, Yamanashi, Kofu, 400-8510 Japan; Department of Physiology, Keio University School of Medicine, 35 Shinanomachi,Shinjuku-ku, Tokyo, 160-8582 Japan; Center for Genomic and Regenerative Medicine, Juntendo University School of Medicine, 2-1-1 Hongo, Bunkyo-ku, Tokyo, 113-8421 Japan; Department of Epigenetic Medicine, Faculty of Medicine, University of Yamanashi, 1110 Shimokato, Chuo, Yamanashi, 409-3898 Japan; Sumitomo Dainipponn Pharma Co. Ltd., Osaka, Osaka, 541-0045 Japan; Department of Neurology,School of Meidicine, Aichi Medical University, 1-1 Yazakokarimata, Nagakute, Aichi, 480-1195 Japan; Department of Dermatology, Keio University School of Medicine, 35 Shinanomachi,Shinjuku-ku, Tokyo, 160-8582 Japan; Department of Stem Cell Biology and Medicine, Graduate School of Medical Sciences, Kyushu University, 3-1-1 Maidashi, Higashi-ku, Fukuoka, 812-8582 Japan

**Keywords:** Rett syndrome, Methyl CpG-binding protein 2/MeCP2, Induced pluripotent stem cell, Neural stem cell, Astrocytes, DNA methylation

## Abstract

**Background:**

Rett syndrome (RTT) is one of the most prevalent neurodevelopmental disorders in females, caused by *de novo* mutations in the X-linked methyl CpG-binding protein 2 gene, *MECP2*. Although abnormal regulation of neuronal genes due to mutant MeCP2 is thought to induce autistic behavior and impaired development in RTT patients, precise cellular mechanisms underlying the aberrant neural progression remain unclear.

**Results:**

Two sets of isogenic pairs of either wild-type or mutant *MECP2-*expressing human induced pluripotent stem cell (hiPSC) lines were generated from a single pair of 10-year-old RTT-monozygotic (MZ) female twins. Mutant *MeCP2*-expressing hiPSC lines did not express detectable MeCP2 protein during any stage of differentiation. The lack of MeCP2 reflected altered gene expression patterns in differentiated neural cells rather than in undifferentiated hiPSCs, as assessed by microarray analysis. Furthermore, MeCP2 deficiency in the neural cell lineage increased astrocyte-specific differentiation from multipotent neural stem cells. Additionally, chromatin immunoprecipitation (ChIP) and bisulfite sequencing assays indicated that anomalous glial fibrillary acidic protein gene (*GFAP*) expression in the MeCP2-negative, differentiated neural cells resulted from the absence of MeCP2 binding to the *GFAP* gene.

**Conclusions:**

An isogenic RTT-hiPSC model demonstrated that MeCP2 participates in the differentiation of neural cells. Moreover, MeCP2 deficiency triggers perturbation of astrocytic gene expression, yielding accelerated astrocyte formation from RTT-hiPSC-derived neural stem cells. These findings are likely to shed new light on astrocytic abnormalities in RTT, and suggest that astrocytes, which are required for neuronal homeostasis and function, might be a new target of RTT therapy.

**Electronic supplementary material:**

The online version of this article (doi:10.1186/s13041-015-0121-2) contains supplementary material, which is available to authorized users.

## Background

Rett syndrome (RTT; MIM 312750) is a representative X-linked neurodevelopmental disease distinguished by repetitive and stereotypic hand movements replacing purposeful hand use, accompanied by gait ataxia, seizures, and autistic features. Patients present with apparently normal psychomotor development during the first 6–18 months of life, followed by a short period of developmental stagnation, and then rapid disease progression with the characteristic features described above [1]. The incidence of RTT is 1 in 10,000–15,000 female births [2,3]. A linkage analysis demonstrated that the region harboring a causative gene is located between Xq27 and Xqter [4]. Meanwhile, a mutation analysis identified an X-linked gene encoding methyl-CpG-binding protein 2 (MeCP2) in the Xq28 region as the cause of RTT, where > ~95% of patients with a classic RTT phenotype carry the *MECP2* mutation [5,6].

MeCP2 is a transcription repressor that inhibits transcription by binding to methylated CpG dinucleotides, and also by recruiting co-repressors and chromatin remodeling proteins [7]. Thus, mutant MeCP2 affects large-scale chromatin organization [8], resulting in the mal-regulation of a number of genes, including neural and synaptic genes [9-13]. Furthermore, because RTT is an X-linked dominant disorder, phenotypic differences between female RTT patients have generally been attributed to variances in X chromosome inactivation (XCI) patterns, with skewing in favor of the mutant allele for the more severe clinical phenotypes [14-16].

Nevertheless, it remains unknown how developmental defects occur in the RTT brain at the cellular level. Recently, human induced pluripotent stem cell (hiPSC) technology has facilitated the modeling of neurological diseases by permitting the reprogramming of somatic cells into pluripotent cells [17]. So far, several studies have been performed with hiPSCs derived from patients with RTT and other neurological and neurodevelopmental diseases [18-26]. Previous reports of differentiated cells derived from RTT patient-specific hiPSCs demonstrated several abnormal *in vitro* phenotypes, such as diminished cell soma and nuclear sizes, reduced expression of neuronal markers, and attenuated dendritic spine density [19,21-24].

We recently reported a rare monozygotic (MZ) case of RTT in twins in which the genomic sequences were identical, including a *MECP2* frame-shift mutation (G269AfsX288) [27]. Interestingly, the patients (designated the RTT-MZ twins) showed divergent symptom severity regarding impaired neurological development, despite an identical genomic structure. Taking advantage of the nonrandom pattern of XCI in female hiPSCs [23] and the shared genetic background of the RTT-MZ twins [27], we aimed to generate two sets of isogenic pairs of wild-type *MECP2-* and mutant *MECP2-*expressing hiPSC lines from the RTT-MZ individuals. We then went on to show detailed defects in neural cells related to MeCP2 protein deficiency-induced developmental defects in RTT. Furthermore, we set out to clarify whether epigenetic and environmental cues participate in the abnormal neural development of RTT patients by comparison of the RTT-hiPSC lines derived from the RTT-MZ twin patients, who presented with varying symptom severity.

## Results

### Mosaic expression patterns in fibroblasts procured from RTT-MZ twins

**Fig. 1:**
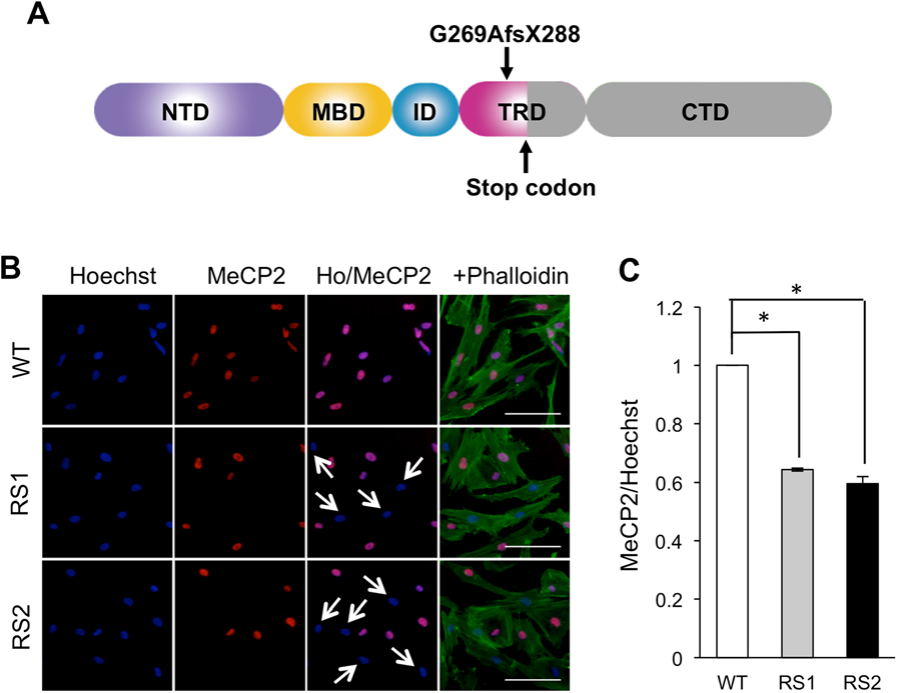
*MECP2* mutation in MZ twins with RTT and MeCP2 expression pattern in RTT fibroblasts. **(A)** Schematic representation of *MECP2* gene structure and location of the *MECP2* mutation. Direct sequencing of the four coding exons in the *MECP2* gene detected a guanine deletion at position 806 (806delG) [27]. **(B)** Immunostaining for MeCP2 (red) and phalloidin (green) along with Hoechst staining (blue) of wild-type *MECP2-* and mutant *MECP2-*expressing fibroblasts. Scale bar, 100 μm. **(C)** Fraction of MeCP2-positive cells among wild-type *MECP2-* and mutant *MECP2-*expressing fibroblasts (n = 5 experiments; > 500 Hoechst-positive cells per experiment; *p < 0.05). WT, wild-type

We recently established fibroblast cell lines from the RTT-MZ twins (patients RS1 and RS2). These RTT twins have a *de novo* frame-shift mutation in exon 4 (c.806delG) that truncates the MeCP2 protein within the transcriptional repression domain (Fig. 1A). We also reported that fibroblasts generated from both patients exhibited random XCI patterns [27], which were detected by the methylation-specific polymerase chain reaction (PCR)-based HUMARA (human androgen receptor) XCI assay [28]. To examine the expression patterns of MeCP2 in RS1 and RS2 fibroblasts, immunostaining was performed with a specific primary antibody against MeCP2. Consequently, the fibroblast lines derived from the RTT-MZ twins included both MeCP2-positive and MeCP2-negative cells (Fig. 1B). Such mosaic expression patterns for the MeCP2 suggests that the fibroblasts comprise MeCP2-positive cells with the X chromosome harboring wild-type *MECP2* as the active MeCP2 species, and MeCP2-negative cells with the X chromosome harboring mutant *MECP2* as the active MeCP2 species. The fractions of MeCP2-positive cells among the RS1 and RS2 fibroblasts were 0.64 and 0.60, respectively (Fig. 1C).

### Generation and characterization of RTT-MZ hiPSC lines

**Fig. 2:**
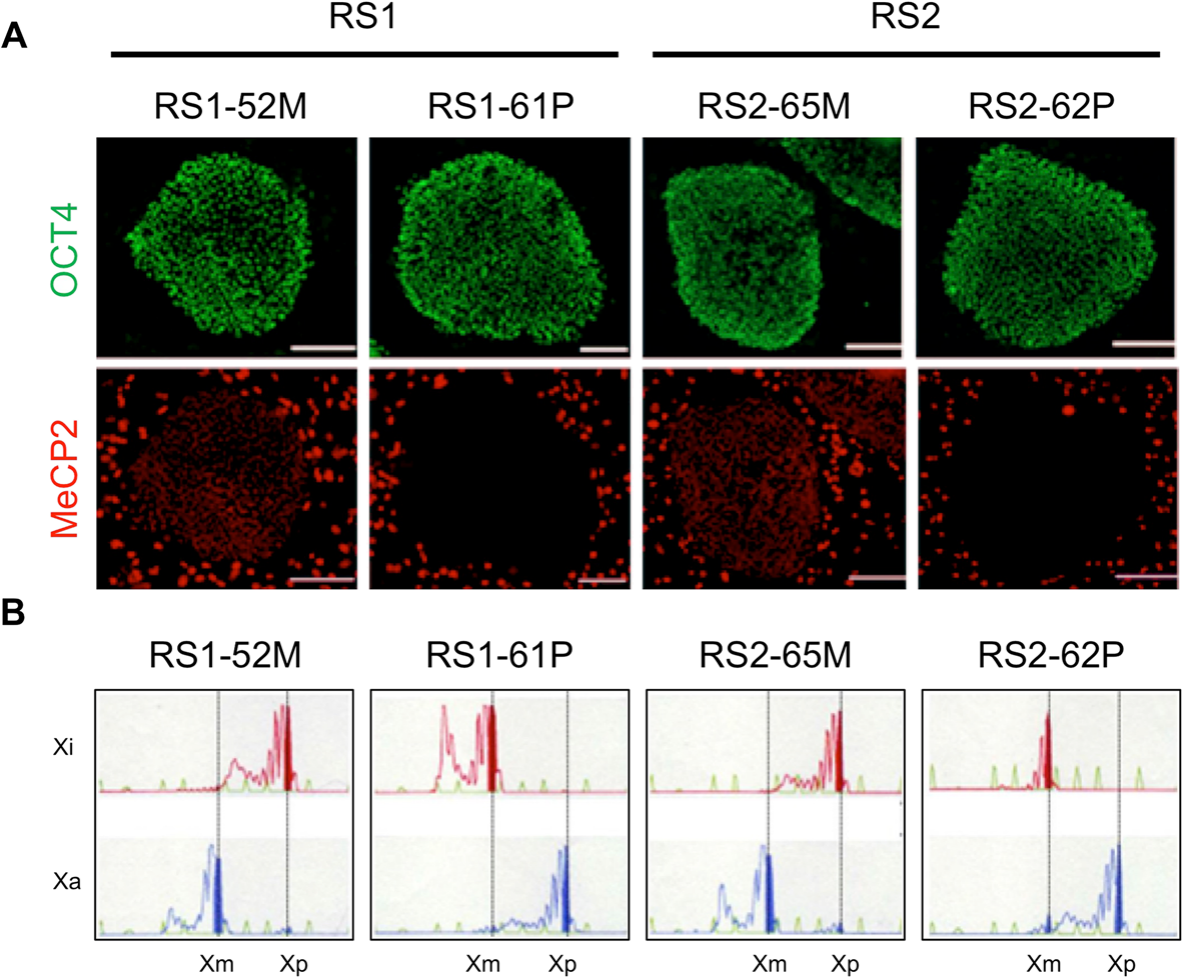
hiPSC lines and XCI patterns derived from the RTT-MZ twins. **(A)** MeCP2 (red) and OCT4 (green) expression in hiPSC lines derived from the RTT-MZ twins. Scale bar, 150 μm. **(B)** XCI patterns in the four hiPSC lines, as assessed by the methylation-specific PCR-based HUMARA assay. Xi, X inactivation pattern based on the inactive X chromosome; Xa, X inactivation pattern based on the active X chromosome; Xm, X chromosome inherited from the mother; Xp, X chromosome inherited from the father

We utilized standard methods and transduction of *OCT4*-, *SOX2*-, *KLF4*-, and *c-MYC*-containing retroviruses to reprogram fibroblasts derived from the RTT-MZ twins into RTT-hiPSCs. Each RTT-hiPSC clone was clonally isolated and selected by morphological criteria and transgene silencing. We also verified pluripotency of the stem cells by immunostaining of the hiPSCs with primary antibodies against pluripotency markers (NANOG, OCT4, and TRA-1-81) (Fig. 2A and Additional file 1A).

To evaluate *in vivo* pluripotency, we injected the RTT-hiPSCs into the testes of immunodeficient mice, and confirmed the formation of teratomas containing derivatives of all three embryonic germ layers (Additional file 1B). No abnormalities were found in the karyotypes of any of the hiPSC lines (Additional file 1C). Notably, most of the selected hiPSC clones were either all MeCP2-positive or MeCP2-negative, and putatively originated from a single MeCP2-positive or MeCP2-negative fibroblast (Fig. 2A). Therefore, we isolated both wild-type *MECP2*-expressing (RS1-52 M and RS2-65 M) and mutant *MECP2*-expressing (RS1-61P and RS2-62P) clones from each patient (RS1 and RS2) to generate four hiPSC lines.

We next analyzed the XCI patterns in the hiPSC lines, and found that the maternal X chromosome was active in the MeCP2-positive (RS1-52 M and RS2-65 M) clones, while the paternal X chromosome was active in the MeCP2-negative (RS1-61P and RS2-62P) clones (Fig. 2B). Although we isolated several clones that partially include iPS cells with two active X chromosomes, we only used clones with an inactive X chromosome (i.e., the “standard” XCI status of undifferentiated hiPSCs [29]) in the present study.

Our previous study [27] revealed that the maternally-derived X chromosome carries the wild-type *MECP2* allele, whereas the paternally-derived X chromosome carries the mutant *MECP2* allele. These results were shown by sequencing the *MECP2* gene in somatic hybrid cell clones carrying either the maternal or the paternal X chromosome of the RTT-MZ twins. Accordingly, the RS1-52 M and RS2-65 M hiPSC lines, in which maternal wild-type *MECP2* was preferentially active, exhibited MeCP2 expression in the nuclei, whereas the RS1-61P and RS2-62P hiPSC lines, in which paternal mutant *MECP2* was preferentially active, did not (Fig. 2A).

### MeCP2 expression in neural cells differentiated from RTT-hiPSCs

**Fig. 3:**
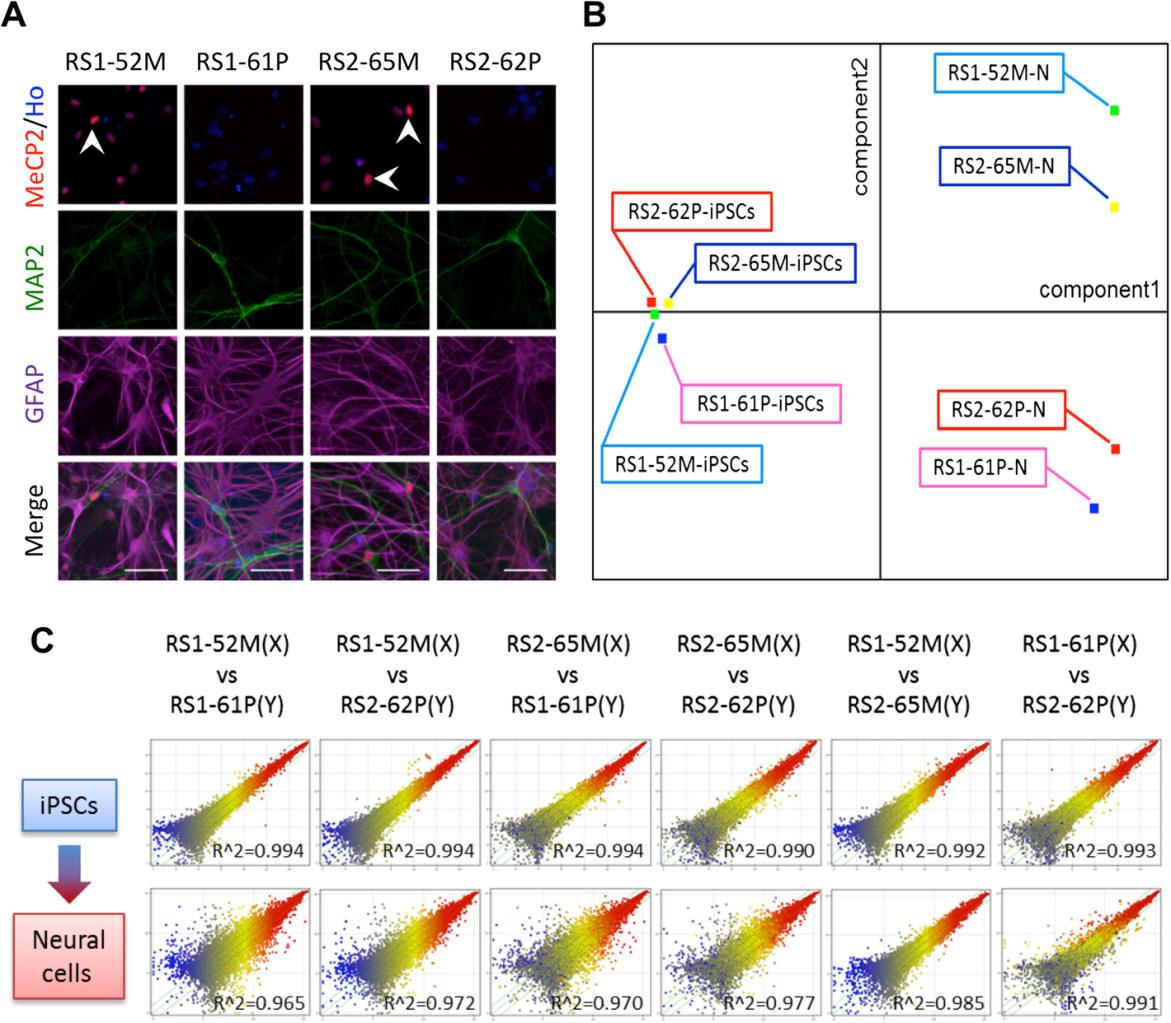
Immunostaining of neural cells derived from RTT-hiPSCs and gene expression analysis of hiPSC-derived neural cells. **(A)** Immunostaining was performed to evaluate expression levels of MeCP2 (red), the neuronal marker, MAP2 (green), and the astrocytic marker, GFAP (magenta) in RTT-hiPSC-derived neural cells. Ho, Hoechst (blue). Arrowhead indicated MAP2, MeCP2 and Hoechst positive cells. Scale bar, 50 *μ*m. **(B)** Results of PCA (performed by using MeV software (TIGR) Software) of microarray gene expression in RTT-hiPSC lines and neural cells differentiated from RTT-hiPSC lines. N, neural cells differentiated from hiPSC lines. **(C)** Scatter plots of microarray gene expression in hiPSC-RTT lines and neural cells differentiated from hiPSC-RTT lines. Neural cells were co-cultured for ~30 days with mouse astrocytes

We next differentiated the four RTT-hiPSC lines into neural cells and examined MeCP2 expression in the progeny. Immunostaining revealed that all of the cells derived from the RS1-52 M and RS2-65 M hiPSC lines (containing preferentially active maternal wild-type *MECP2*) expressed MeCP2 in the nucleus, like the parental hiPSCs. In particular, MAP2-positive neurons expressed MeCP2 more strongly than glial fibrillary acidic protein (GFAP)-positive astrocytes (Fig. 3A) as previously shown in mice [30,31]. However, no MeCP2 expression was found in the progeny of the RS1-61P and RS2-62P hiPSC lines (containing preferentially active paternal mutant *MECP2*), or in the nucleus of RS1-61P/RS2-62P hiPSC-derived MAP2-positive neurons or GFAP-positive astrocytes (Fig. 3A). Therefore, neural cells differentiated from the RTT-hiPSC lines apparently retain the XCI status of the undifferentiated cells.

### Global gene expression in RTT-hiPSCs and differentiated neural cells

We next investigated whether the loss of MeCP2 protein affects the transcriptional network in undifferentiated hiPSCs and neural progeny differentiated from RTT-hiPSCs. Global gene expression levels in wild-type *MECP2*- and mutant *MECP2*-expressing cells were examined by comparative microarray analyses of undifferentiated hiPSCs and differentiated neural cells. The gene expression data were normalized and subjected to principal component analysis (PCA) and hierarchical clustering, with the exception of those genes with low expression in all samples.

The samples were clustered into hiPSC or neural cell (N) groups depending on the *MECP2* expression pattern. In the PCA analysis, the original hiPSCs were clustered tightly into one region, signifying few differences from one hiPSC clone to the next. On the other hand, the neural cells were roughly clustered into two groups that reflected the status of the activated X chromosome (Fig. 3B and Additional file 2). Next, we constructed scatter plots to compare wild-type *MECP2*- and mutant *MECP2*-expressing cells. In the undifferentiated hiPSCs, the correlation coefficient (R^2^) was > 0.99 for any pair of hiPSC clones. However, in the differentiated neural cells, higher correlations (R^2^ > 0.98) were only found in comparisons of hiPSCs with the same XCI pattern (i.e., paternal X vs. paternal X or maternal X vs. maternal X), while lower correlations (R^2^ < 0.98) were only found in comparisons of hiPSCs with different XCI patterns (paternal X vs. maternal X) (Fig. 3C). The clustering dendrogram (Additional file 3A) also showed that global gene expressions for all four RTT-hiPSC clones during the undifferentiated stage. After this time, mutant *MECP2*-expressing hiPSCs became distinguishable from wild-type *MECP2*-expressing hiPSCs due to neural differentiation. Nevertheless, Tanaka et al. [25] recently characterized five patient-speific cell lines with different mutations to show that hiPSCs with *MECP2* gene mutations are distinguishable from normal hiPSCs and ESCs even during the undifferentiated stage. By contrast, our isogenic cell lines might largely exclude the effect of donor divergence, permitting the elucidation of *MECP2* function during neural differentiation. While Tanaka and colleagues [25] demonstrated that the gene expression of a key mitochondrial transcription factor, *NR3C1*, was upregulated in mutant *MECP2*-expressing cells after neuronal differentiation, we failed to detect such a difference in the current study (Additional file 3B). These results suggest that MeCP2 plays a more important role as a transcriptional regulator in differentiated neural cells than in undifferentiated hiPSCs.

### Enhanced astrocytic differentiation of mutant *MECP2*-expressing neural stem cells derived from RTT-hiPSCs

**Fig. 4:**
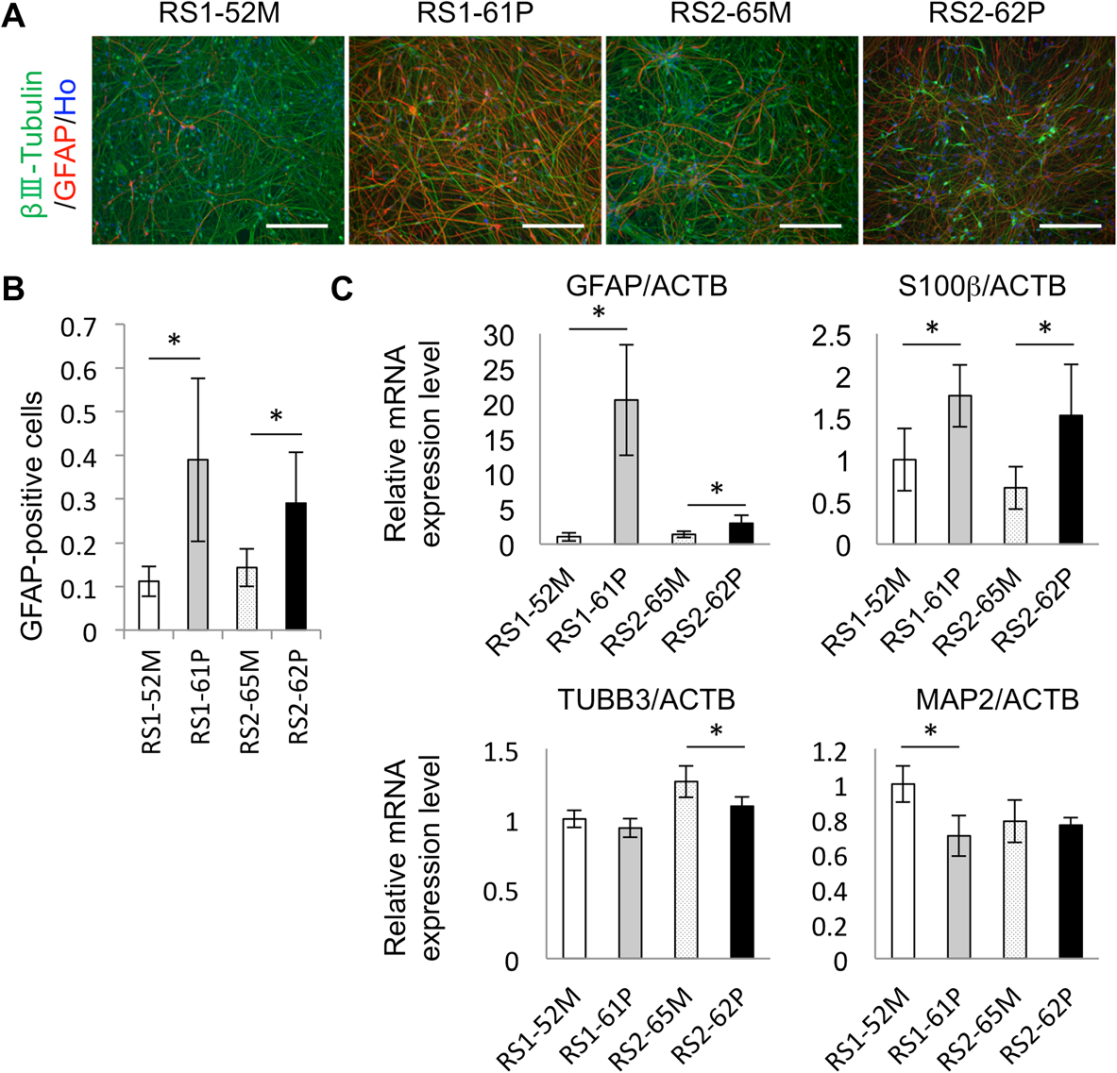
Comparison of acquired neuronal and astrocytic properties between MeCP2-positive and negative hiPSC-derived neural cells. **(A)** Immunostaining images of neural cells. Double labeling for GFAP (red) and βIII-tubulin (green) is shown along with Hoechst staining (Ho, blue) in RS1-52 M, RS1-61P, RS2-65 M, and RS2-62P RTT-hiPSC-derived neurons. Scale bar, 200 *μ*m. **(B)** Fraction of GFAP-positive cells relative to Hoechst-positive cells in neural cells differentiated from RTT-hiPSC lines. **(C)** A qPCR-facilitated comparison of gene expression for astrocytic markers (*GFAP*, *S100β*) and neuronal markers *(TUBB3, MAP2*) in neural cells differentiated from RTT-hiPSC lines. Relative gene expression levels were normalized to that of *ACTB.* Data in **(B)** and **(C)** were analyzed by Student’s *t*-test and Welch’s *t*-test (*p < 0.05)

Previous studies revealed that MeCP2 is involved in the regulation of astroglial gene expression [32-34]. *Gfap* and *S100β* are expressed at significantly higher levels in astrocytes derived from MeCP2-null mouse embryonic stem cells than in those derived from wild type mouse embryonic stem cells [35,36]. Moreover, a truncated form of RTT-associated MeCP2 (R168X) is reported to be unable to promote neuronal differentiation in mice, and instead promotes an abnormally high degree of astrocytic differentiation [37]. We characterized neurospheres derived from the four RTT-hiPSC lines (RS1-61P, RS2-62P, RS1-52 M, and RS2-65 M), and found no significant differences in neurosphere number, size, or expression of neural stem cell markers between the two groups derived from mutant vs. wild-type *MECP2*-expressing hiPSCs (Additional file 4). Next, the neurospheres were differentiated into neural cells by using an adhesion culture method without fibroblast growth factor-2 (FGF-2) at ~30 days *in vitro* (Fig. 4A). The differentiated cells were classified by their expression of βIII-tubulin and GFAP. Immunocytochemical analysis revealed that the neural cells originating from MeCP2-negative hiPSC lines (RS1-61P and RS2-62P) contained significantly higher proportions of GFAP-positive cells than those originating from MeCP2-positive hiPSC lines (RS1-52 M and RS2-65 M) (Fig. 4B). Most of the GFAP-positive cells failed to express neuronal markers; therefore, we concluded that these cells were in fact astrocytes.

During the neural development of the mammalian central nervous system (CNS), neural stem cells initially differentiate into neurons, followed by astrocytes and oligodendrocytes at a later stage. We and others reported that MeCP2 binds to the promoter region of astrocyte-specific genes, including *S100β* and *Gfap*, to inhibit the conversion of neurons into astrocytes in mammals [33,34,37-39]. To determine how MeCP2-regulated astrocyte-specific genes are influenced by the absence of MeCP2, we next examined the expression of *S100β* and *GFAP* in MeCP2-negative and McCP2-positive neural cells. As a result, MeCP2-negative RS1-61P and RS2-62P neural cells showed significantly enhanced expression of the astrocyte-specific genes, *GFAP* and *S100β* (Fig. 4C). However, decreased expression levels of the neuronal genes, *TUBB3* and *MAP2*, were observed in several samples (i.e., *TUBB3* in RS2-62P cells and *MAP2* in RS1-61 cells) (Fig. 4C). These observations suggest that MeCP2 deficiency in the neural cell lineage increases astrocytic differentiation from multipotent neural stem cells.

### Lack of MeCP2 binding to the *GFAP* gene in RTT-hiPSC-derived neural stem cells

**Fig. 5:**
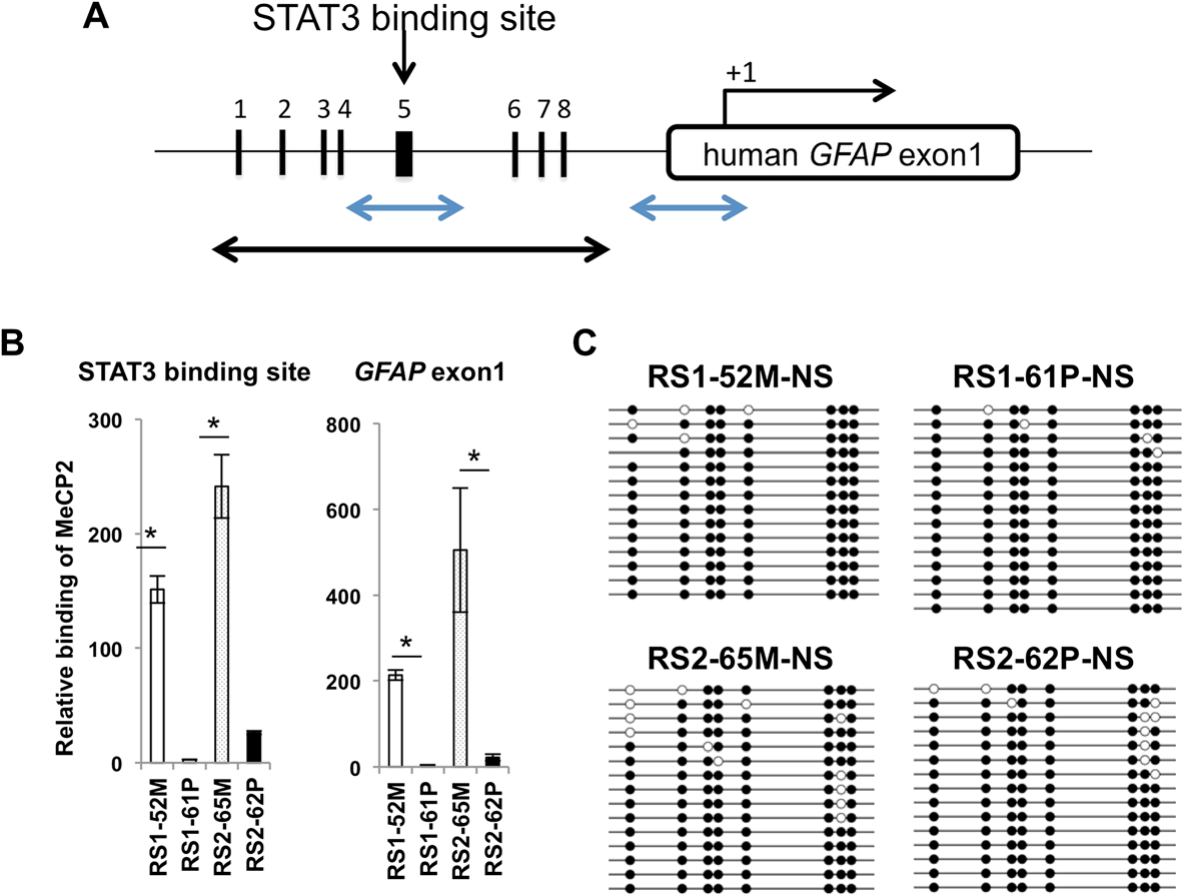
MeCP2 binding analysis and DNA methylation status of the *GFAP* promoter region. **(A)** Schematic representation of the *hGFAP* genomic locus. ChIP/qPCR analysis was performed for the genomic regions (i.e., the STAT3 binding site and *GFAP* exon 1) indicated by the blue bidirectional arrows. Bisulfite sequencing was performed for the genomic region indicated by the black bidirectional arrows. **(B)** MeCP2 binding to the STAT3 binding site within the *GFAP* promoter region was quantified via ChIP/qPCR analysis by using an anti-MeCP2 antibody in neural cells differentiated from RTT-hiPSC lines. **(C)** Methylation frequencies of (1) the CpG site within the STAT3 recognition sequence and (2) seven other CpG sites around this sequence were analyzed in hiPSC-derived neurosphres via bisulfite sequencing

MeCP2 was previously reported to bind to the highly methylated exon 1 region of the *Gfap* gene in mouse neurons [33,38]. To confirm these results in wild-type or mutant *MECP2*-expressing neurospheres, we performed chromatin immunoprecipitation (ChIP) assays with a specific primary antibody against MeCP2. Binding data were quantitated via real-time PCR by using primers spanning from the STAT3 binding site within the *GFAP* promoter to exon 1, relative to the transcription start site of *GFAP* (Fig. 5A, indicated by blue bidirectional arrows). For both the STAT3 binding site and *GFAP* exon 1, precipitated genomic fragments were scarcely detectable by quantitative PCR (qPCR) in neurospheres derived from mutant *MECP2*-expressing hiPSC lines (RS1-61P, RS2-62P), while significant precipitation of genomic fragments was detected in neurospheres derived from wild-type *MECP2*-expressing hiPSC lines (RS1-52 M and RS2-65 M) (Fig. 5B).

Based on these results, we hypothesized that the increased expression of GFAP was induced by lack of direct MeCP2 binding to the *GFAP* gene in the mutant *MECP2*-expressing cells. However, it is possible that MeCP2 dysfunction indirectly enhanced the maturation of neural stem cells with truncated MeCP2, accompanied by decreased methylation of *gfap* [33], which might in turn modify MeCP2 protein recruitment. Thus, we performed bisulfite sequencing at the STAT3 binding site within the *GFAP* locus (Fig. 5A, indicated by black bidirectional arrows) by using wild-type or mutant *MECP2*-expressing neurospheres. Nevertheless, the MeCP2 binding sites in the *GFAP* gene were similarly hypermethylated in both wild-type and mutant *MECP2*-expressing neural stem cells differentiated from the four hiPSC lines (Fig. 5C). These findings signify that aberrant *GFAP* gene expression in neural stem cells containing truncated MeCP2 is not caused by changes in DNA methylation status (i.e., hypomethylation) within the genomic region encompassing the STAT3 binding site. Instead, the aberrant gene expression may be due to alterations in the amount of MeCP2 that binds to the STAT3 binding site within the *GFAP* promoter and the *hGFAP* exon1.

## Discussion

Female RTT patients display somatic mosaicism, because the *MECP2* gene is located on the X chromosome and undergoes random XCI. Here, we successfully generated two kinds of isogenic RTT-hiPSC lines that expressed only the wild-type or the mutant *MECP2* allele from the same RTT patient. So far, several groups have demonstrated disease modeling of RTT by using patient-specific hiPSCs [19,21-24]. However, some of these studies were conducted by comparisons between mutant hiPSCs derived from RTT patients and control hiPSCs derived from healthy subjects [19].

Regarding hiPSC technologies, clonal variations caused by donor differences and cell types of origin reportedly determine the differentiation potential of hiPSC clones. Hence, isogenic controls genetically engineered by using ZFN, TALEN or CRISPR-Cas9 systems are required for precise disease modeling with patient-specific hiPSCs [40]. Our isogenic RTT-hiPSC system has two significant advantages over these previous systems. First, our system allows clonal generation of hiPSCs expressing either wild-type or mutant MeCP2 protein. Second, the generated hiPSCs are isogenic without genetic engineering. By utilizing our homogenous RTT-hiPSC populations, we were able to show that global gene expression patterns were distinctly different between wild-type MeCP2-cells and mutant MeCP2-expressing cells in terms of both neurospheres and differentiated neural stem cells. Although MeCP2 was weakly expressed in undifferentiated wild-type hiPSCs, significant differences in gene expression profiles were not observed among the four hiPSC lines. These results suggest that the MeCP2 primarily functions in cells already committed to a neural lineage.

We also found increased proportions of astrocytes among mutant MeCP2-expressing neural cells compared with wild-type MeCP2-expressing neural cells. Other investigators similarly documented MeCP2 expression in glial cells, including astrocytes [41], implying that glial MeCP2 might play a distinct role in the maintenance of neuronal functions, such as neuronal maturation and/or dendritic arborization [42]. Although previous studies showed that a conditional MeCP2 knockout mouse (which lacked MeCP2 expression in mature neurons) displayed a RTT-like neurological phenotype, mutant astrocytes derived from this model mouse failed to support normal dendritic arborization of either wild-type or mutant neurons [30]. Furthermore, mutant *MECP2*-expressing astrocytes differentiated from RTT-hiPSCs apparently had significant effects on wild-type neurons [26]. Intriguingly, another study in mice showed that the MeCP2-deficient state can spread between astrocytes via gap junctions [43].

The current investigation did not examine dendritic phenotypes in neurons derived from RTT-hiPSCs. However, our model may be useful for mixed culture experiments (i.e., wild-type *MECP2*-expressing neurons and mutant *MECP2-*expressing astrocytes) to distinguish cell-autonomous from non-cell-autonomous effects in RTT patient-derived neural cells. Additionally, we demonstrated that astrocytic marker genes, particularly *GFAP*, were aberrantly expressed under conditions of MeCP2 deficiency due to the deregulation of transcription, and we employed ChIP and bisulfite sequencing analyses to show induction of GFAP expression in RTT-hiPSC-derived neural cells caused by the absence of direct binding of mutant MeCP2 to the *GFAP* gene. Hence, mutant MeCP2 may contribute to the excessive differentiation of GFAP-positive astrocytes.

Several groups reported that human female RTT patients present with microcephalic brains, as do male and female Rett syndrome model mice [44-46]. Moreover, analysis of postmortem brain tissue from RTT patients revealed increased GFAP protein expression in the RTT brain [32]. Consistent with these results, we anticipate that the brains of RTT patients will exhibit a larger than normal percentage of astrocytes. Thus, astrocytes differentiated from RTT patient-derived, mutant *MECP2*-expressing hiPSC lines may be useful for drug development for RTT therapy, because some drugs for mental disorders (e.g., antidepressants) affect not only neurons, but also activate astrocytes. The activated astrocytes then go on to carry out specific functions that result in the reactivation of cortical plasticity, potentially leading to the readjustment of abnormal neural networks [47,48].

This study aimed to clarify the involvement of epigenetic and environmental cues in the neural development of RTT-MZ twins (patients RS1 and RS2) with differing symptom severity. Although we successfully generated hiPSCs from both patients, no significant differences in mutant MeCP2 phenotypes were observed between RS1 and RS2 cells. These results suggest that epigenetic and environmental cues might work together to affect the neural phenotypes of RTT patients. Another possibility is that skewed XCI occurs in the CNS in RTT patients. Although we previously reported that biased XCI does not take place in the other somatic cells (i.e., skin, blood, and hair) of these patients [27], biased XCI in the CNS may underlie the discrepancy in symptom severity between isogenic RTT-MZ twin patients.

## Conclusions

In summary, we successfully generated two sets of isogenic RTT-hiPSC lines that expressed only the wild-type or the mutant *MECP2* allele from the same RTT patient, and revealed that abnormal astrocytic differentiation is involved in RTT pathogenesis. Specifically, we used hiPSC technology to show that (1) MeCP2 protein deficiency results in dysregulation of *GFAP* expression in mutant *MECP2*-expressing neural cells, and (2) an increased number of astrocytes are differentiated from RTT-hiPSC-derived neural stem cells carrying mutant vs. wild-type *MECP2*.

## Materials and methods

### Isolation of skin fibroblasts and generation of hiPSCs

A skin-punch biopsy from a single pair of 10-year-old Japanese MZ twins with RTT (patients RS1 and RS2) [27] was performed with written informed consent from their parents. The obtained fibroblasts were used to establish the four hiPSC lines discussed above. The study protocol was reviewed and approved by the Research Ethics Committees of Keio University (Approval No. 20080016) and the University of Yamanashi (Approval Nos. 523 and 699). This study was conducted in accordance with the principles expressed in the Declaration of Helsinki.

A standard retrovirus method was used to establish the hiPSCs. The maintenance of fibroblasts, lentiviral production, retroviral production, infection, stem cell culture, and characterization of hiPSCs was conducted as described previously [17].

### Sample collection and DNA isolation

All peripheral blood, skin fibroblasts, and hiPSC samples used in this study were obtained from RTT-MZ patients whose parents gave informed consent for medical research at the University of Yamanashi and Keio University Hospital. DNA from lymphocytes, skin fibroblasts, and hiPSCs was extracted with the DNeasy Blood and Tissue Kit (QIAGEN) according to the manufacturer’s instructions.

### XCI analysis

XCI patterns were obtained in the extracted DNA by using a methylation-specific PCR-based method. Bisulfite treatment of genomic DNA followed by PCR was performed as previously described [28]. Briefly, DNA was treated with sodium bisulfite, and the treated DNA was amplified via PCR by using two primer sets (one for the methylated inactive X chromosome, and the other for the unmethylated active X chromosome). The primer sets were designed within the CpG island (exon 1) of the HUMARA gene located at Xq11-a12. The PCR products were mixed with a size standard and separated on an ABI 310 DNA Sequencer equipped with Genescan Software (Applied Biosystems).

### Teratoma assay

Teratoma formation was assessed by injecting hiPSCs into the testes of 8-week-old NOD.CB17-*Prkdc*^scid^/J mice (OYC International, Inc.), as previously described [49]. Eight weeks after hiPSC transplantation, tumors were dissected and fixed with phosphate buffered saline (PBS) containing 4% formaldehyde. Paraffin-embedded tissue was sectioned and stained with hematoxylin & eosin. Images were obtained by using a BZ-9000 microscope (Keyence).

### Karyotyping of hiPSCs

Standard G-banding analysis was performed in the four hiPSC lines (RS1-52 M, RS1-61P, RS2-65 M, and RS2-62P) to rule out the possibility of abnormal karyotypes that can occur during the generation of hiPSCs.

### *In vitro* differentiation of hiPSCs

Neural differentiation of hiPSCs was performed as described previously [50], except that epidermal growth factor and Noggin were omitted from the culture system (Matsumoto et al., manuscript in submission). The hiPSCs were plated onto the bottom of T75 flasks (Nunclon) and maintained for 14 days. Neurospheres were repeatedly passaged by dissociation into single cells, followed by culture in the same manner. Neurospheres were differentiated into neural cells by using an adhesion culture method without FGF-2, and then subjected to qPCR and immunostaining analysis. Typically, neurospheres between passages 3 and 10 were used for the analysis. RNA samples were collected on day 14 of neurosphere culture from an arbitrary number of cells. For terminal differentiation, neurospheres were cultured in 1 × B27 medium, dissociated neural precursor cells allowed to adhere to poly-L-ornithine-/fibronectin-coated coverslips and then cultured for 30–32 days. RNA was then isolated from terminally differentiated neurospheres. Alternatively, the differentiated cells were immunostained with primary antibodies against βIII-tubulin and GFAP. Approximately 1 × 10^5^ neural stem cells were plated onto 14-mm glass coverslips for immunostaining of differentiated neural cells, and ~ 1.2 × 10^6^ neural stem cells were plated onto a well using 6-well plate for RNA isolation from differentiated neural cells. For long-term neural differentiation, mouse astrocytes obtained from E16.5 ICR mice were utilized with a cell culture insert system (Falcon® product #353102). RNA samples were obtained from iPSCs, neurospheres, and neuronal cells at passages 12–20, passages 3–9 (day 14), and day 30–32, respectively.

### Immunocytochemical analysis of hiPSCs and neural cells differentiated from hiPSCs

Cells were fixed with PBS containing 4% paraformaldehyde for 30 min at room temperature and incubated with primary antibodies against the following proteins: MeCP2 (1:200, Cell Signaling Technology), phalloidin (1:2000, Dyomics), NANOG (1:500, CosmoBio), OCT4 (1:200, Santa Cruz Biotechnology, Inc.), TRA-1-60 (1:1000, Millipore), TRA-1-81 (1:1000, Millipore), βIII-tubulin (1:2000, Sigma Chemical Co.), MAP2 (1:250, Sigma Chemical Co.), and GFAP (1:1000, Invitrogen). They were then washed with PBS and incubated with an Alexa Fluor 488-, Alexa Fluor 555-, or Alexa Fluor 647-conjugated secondary antibody (1:500, Invitrogen), as appropriate. Images were obtained using an Axioplan 2 microscope (Carl Zeiss). The number of GFAP-positive cells was counted among 100 Hoechst-positive cells for each experiment (n = 5).

### Microarray analysis

For microarray analysis, RNA quality was assessed by using a 2100 Bioanalyzer (Agilent Technologies). Total RNA (100 ng) was reverse transcribed, biotin- labeled, and hybridized to a GeneChip® Human Genome U133 plus 2.0 Array (Affymetrix). The array was subsequently washed and stained in a Fluidics Station 450 (Affymetrix), as instructed by the manufacturer. The microarrays were scanned by using a GeneChip® Scanner 3000 (Affymetrix), and the raw image files were converted into normalized signal intensity values by using the MAS 5.0 algorithm.

Next, targets were selected that (a) were “present” in at least one of the eight arrays analyzed, and (b) had a probe intensity of ≥ 50. In total, 23,463 targets were identified from this initial screen. The normalized logs were hierarchically clustered based on uncentered correlation with complete linkage by using Cluster 3.0 Software [51]. The PCA plot was generated by using TIGR MeV (Multiple Experimental Viewer) Software [52]. The three-dimensional image of the PCA plot and the scatter plot were generated by using GeneSpring GX Software (ver. 12.6.1).

### Quantitative reverse transcription PCR (qPCR) assay

Total cellular RNA was extracted by using the TRIzol Reagent® (Life Technologies), the RNase-Free DNase Set (QIAGEN), and the RNeasy® Mini Kit (QIAGEN). Next, cDNA synthesis was performed by using the SuperScript III First-Strand Synthesis System for RT-PCR (Life Technologies) with oligo-dT primers according to the manufacturer’s guidelines. Real-time RT-PCR was then performed on a ABI PRISM® 7900HT Sequence Detection System (Applied BioSystems) by using SYBR Premix ExTaq Tli RNaseH Plus (Takara). The qPCR amplification was performed by using the following primers: *GFAP*-forward (5’-TGTGAGGCAGAAGCTCCAGGATGA-3’) and *GFAP*-reverse (5’- AGGGTGGCTTCATCTGCTTCCTGT-3’); *S100β*-forward (5’-GTGGCCCTCATCGACGTTTT-3’) and *S100β*-reverse (5’-ACCTCCTGCTCTTTGATTTCCTCT-3’); *TUBB3*-forward (5’-ATTTCATCTTTGGTCAGAGTGGGGC-3’) and *TUBB3*-reverse (5’-TGCAGGCAGTCGCAGTTTTCAC-3’); *MAP2*-forward (5’-GGCCCAAGCTAAAGTTGGTTCTC-3’) and *MAP2*-reverse (5’-GCAGTGACATCCTCAGCCAAAG-3’); and *ACTB*-forward (5’-TGAAGTGTGACGTGGACATC-3’) and *ACTB*-reverse (5’-GGAGGAGCAATGATCTTGAT-3’). Relative gene expression levels of *GFAP*, *S100β*, *TUBB3,* and *MAP2* were normalized to *ACTB* expression and standardized by the RS1-52 M value, which was set to 1.

### ChIP assay

Nuclei were obtained from neurospheres derived from each hiPSC line. ChIP was performed with antibodies against MeCP2 (Cell Signaling Technology), as described previously [53], with the following modifications. Neurospheres were crosslinked with 1% formaldehyde for 10 min to yield crosslinked chromatin. They were then incubated with glycine at a final concentration of 200 mM for another 5 min, and stored at −80°C until use. The lysed nuclear pellets were sonicated six times with a 30-sec ON, 60-sec OFF cycle by using a Bioruptor® Sonicator (Diagenode, Inc.). The crosslinked chromatin was subsequently eluted from magnetic beads (Dynabeads® M-280 Sheep anti-Rabbit IgG; Life Technologies). Co-immunoprecipitated DNA was detected via qPCR by using the following primers: STAT3 binding site-forward (5’-TCATGCCCAGTGAATGACTC-3’) and STAT3 binding site-reverse (5’-AGATGCCAGGCTGTCAGG-3’); and *hGFAP* exon1-forward (5’-AGAGCAGGATGGAGAGGAGA-3’) and *hGFAP* exon1-reverse (5’- CCTTGAAGCCAGCATTGAGT-3’).

### Bisulfite sequencing analysis

Sodium bisulfite treatment of genomic DNA was performed by using the Methylamp DNA Modification Kit (Epigentek) according to the manufacturer’s instructions. A region in the *GFAP* promoter region containing the STAT3 binding site was amplified from bisulfite-treated genomic DNA via PCR with the following forward and reverse primers: *hGFAP* promoter-forward (5’-TTGGGGAGGAGGTAGATAGTTAGGTTTT-3’) and *hGFAP* promoter-reverse (5’-CATCCCCTAATCCCCTTTCCTAAA-3’). PCR products were cloned into the pT7Blue vector (Novagen), and at least 12 randomly selected clones were sequenced.

### Statistical analysis

All quantifiable data are expressed as the means ± the standard deviation of the mean. The statistical significance of differences between conditions was analyzed by using Student’s *t*-test and Welch’s *t*-test. In all cases, *p* values of < 0.05 were considered statistically significant.

## Additional files

Additional file 1: **Characterization of RTT-hiPSCs.** **(A)** Isogenic RTT-hiPSCs demonstrate similar embryonic stem cell-like morphology and stain positively for the pluripotency markers, NANOG, OCT4, and TRA-1-81. Scale bar for phase contrast images, 500 μm; scale bar for NANOG, OCT4, and TRA-1-81 immunostaining, 50 μm. **(B)** Representative images of teratomas generated in immunodeficient mice that received an intratesticular injection of RTT-hiPSCs. The teratomas corresponded to well-defined, cystic tumors containing tissues of all three germ layers (endoderm, mesoderm, and ectoderm). Scale bar, 100 *μ*m. **(C)** Images of RTT-hiPSC karyotypes. (PDF 246 kb)

Additional file 2: **Three-dimensional image of the PCA.** The clustering pattern of the cells (hiPSCs vs. neural cells (N) was dependent on the *MECP2* expression pattern and the presence or absence of MeCP2 protein.

Additional file 3: **Gene expression analysis of iPSCs.** **(A)** Global gene expression/comparative microarray analyses of undifferentiated hiPSCs and differentiated neural cells. N denotes neural cells. **(B)** Comparison of *NR3C1* gene expression in hiPSCs and differentiated neural cells by qPCR. Relative gene expression was normalized by *ACTB* gene expression. Primer sets are listed in Additional file 5. Data were analyzed by Student’s *t*-test and Welch’s *t-*test (*p < 0.05).

Additional file 4: **Properties of neural stem cells derived from RTT-hiPSCs.** **(A)** Diameter (n = 3) and **(B)** number of neurospheres (n = 16) derived from RTT-hiPSCs at 7 days *in vitro*. **(C)** The mRNA expression levels of the neural stem cell markers, *NESTIN* (NES) and *SOX1*, were quantified in RTT hiPSC-derived neurospheres by qPCR. Each value was normalized by comparison with *ACTB* expression and standardized by the value for the wild-type *MECP2*-expressing RS1-52 M clone, which was set to 1 (n = 4). Primer sets are listed in Additional file 5. Data were analyzed by Student’s *t*-test and Welch’s *t*-test (*p < 0.05).

Additional file 5: **List of primers used in Additional file 3 and 4.**
