# Supplementary figures and images for: Differentiation of multipotent neural stem cells derived from Rett syndrome patients is biased toward the astrocytic lineage
Source: Mol Brain. 2015 May 27;8:31. doi: 10.1186/s13041-015-0121-2 (PMC4446051; doi:10.1186/s13041-015-0121-2)

**A**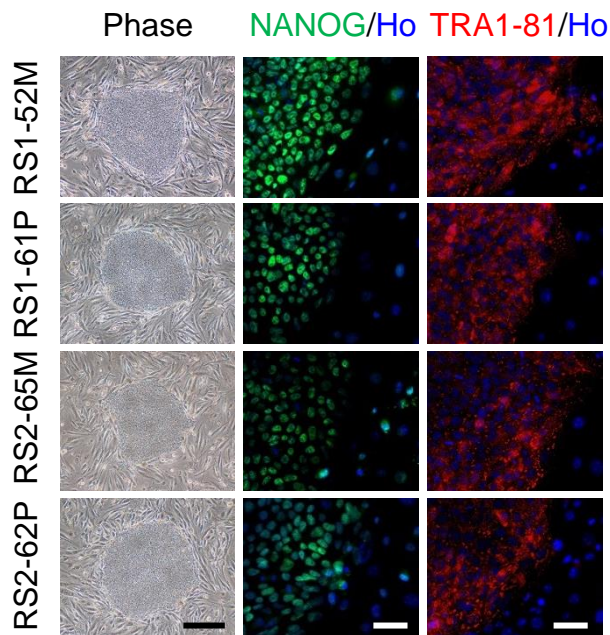**B**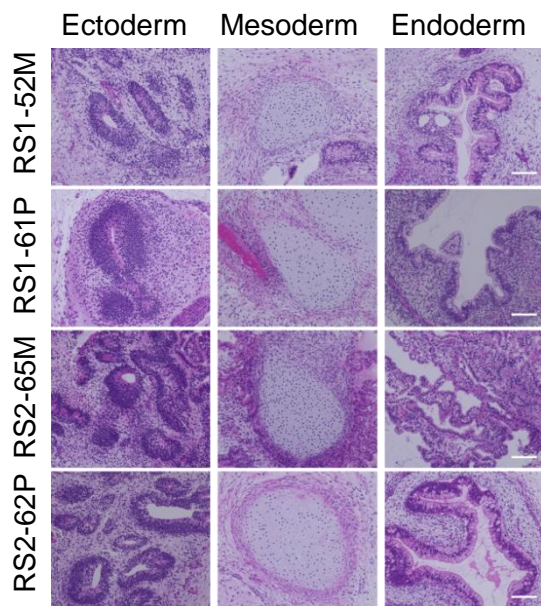**C**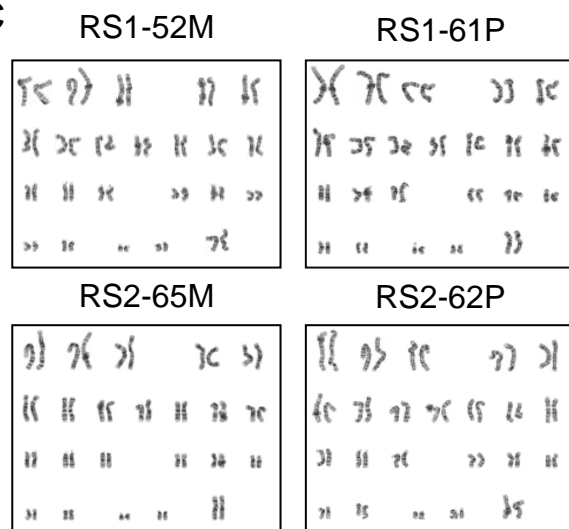

Supplement: Supplementary file 1 — Characterization of RTT-hiPSCs. (A) Isogenic RTT-hiPSCs demonstrate similar embryonic stem cell-like morphology and stain positively for the pluripotency markers, NANOG, OCT4, and TRA-1-81. Scale bar for phase contrast images, 500 μm; scale bar for NANOG, OCT4, and TRA-1-81 immunostaining, 50 μm. (B) Representative images of teratomas generated in immunodeficient mice that received an intratesticular injection of RTT-hiPSCs. The teratomas corresponded to well-defined, cystic tumors containing tissues of all three germ layers (endoderm, mesoderm, and ectoderm). Scale bar, 100 μm. (C) Images of RTT-hiPSC karyotypes. (PDF 246 kb) [file 13041_2015_121_MOESM1_ESM.pdf]

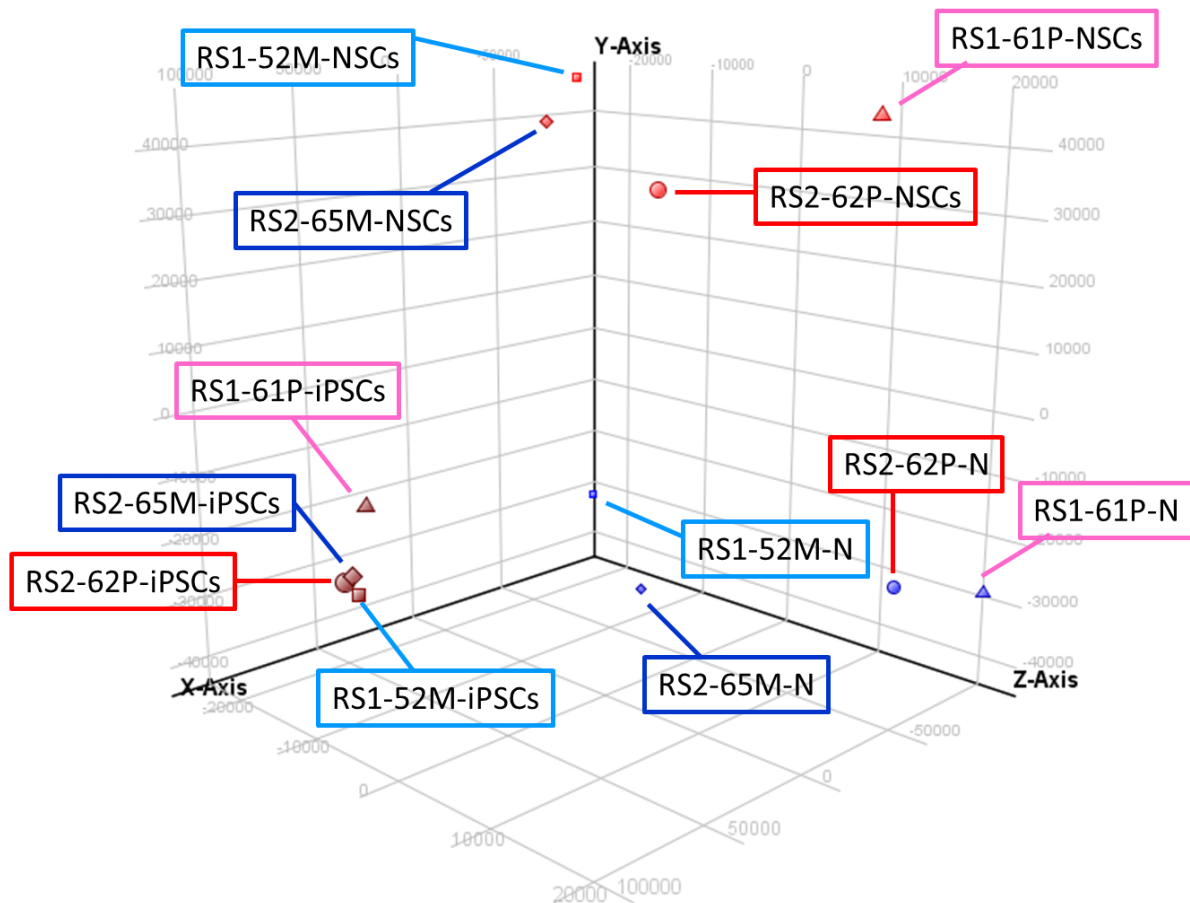

Supplement: Supplementary file 2 — Three-dimensional image of the PCA. The clustering pattern of the cells (hiPSCs vs. neural cells (N) was dependent on the MECP2 expression pattern and the presence or absence of MeCP2 protein. [file 13041_2015_121_MOESM2_ESM.pdf]

**A**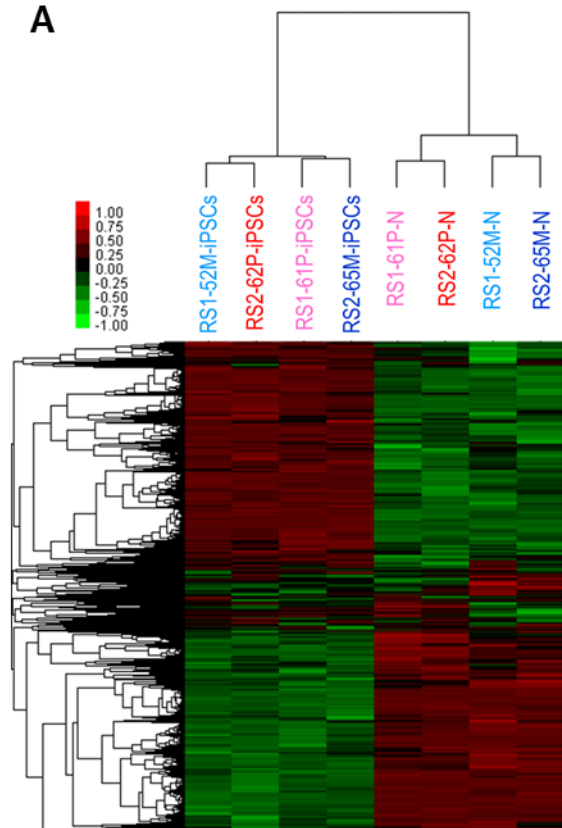**B**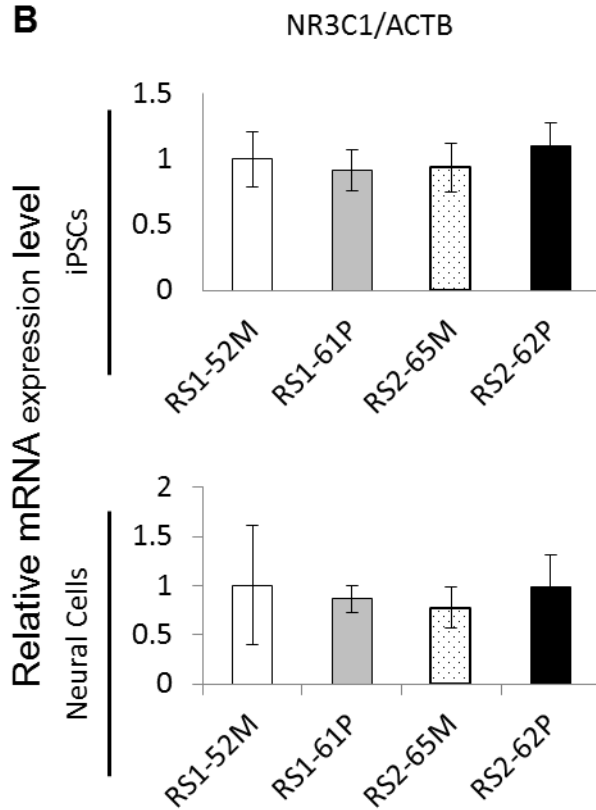

Supplement: Supplementary file 3 — Gene expression analysis of iPSCs. (A) Global gene expression/comparative microarray analyses of undifferentiated hiPSCs and differentiated neural cells. N denotes neural cells. (B) Comparison of NR3C1 gene expression in hiPSCs and differentiated neural cells by qPCR. Relative gene expression was normalized by ACTB gene expression. Primer sets are listed in Additional file 5. Data were analyzed by Student’s t-test and Welch’s t-test (*p < 0.05). [file 13041_2015_121_MOESM3_ESM.pdf]

**A**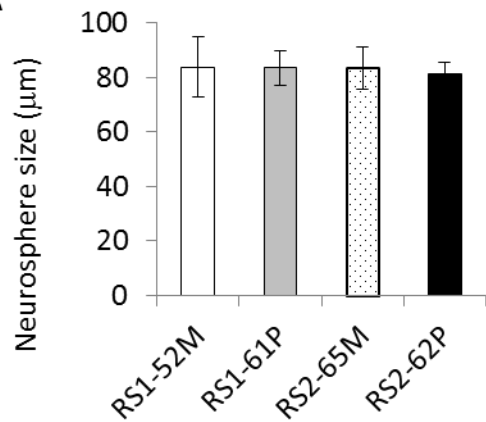**B**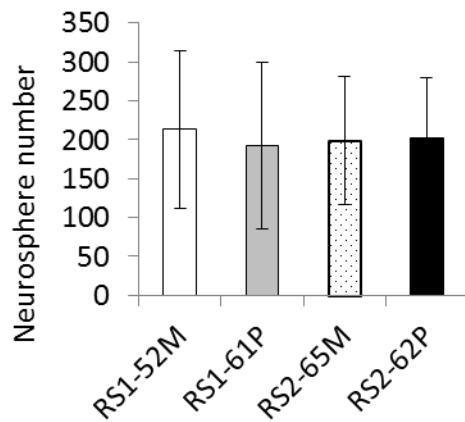**C**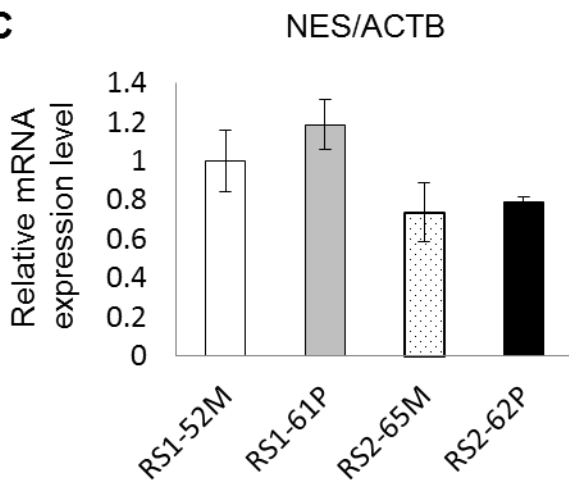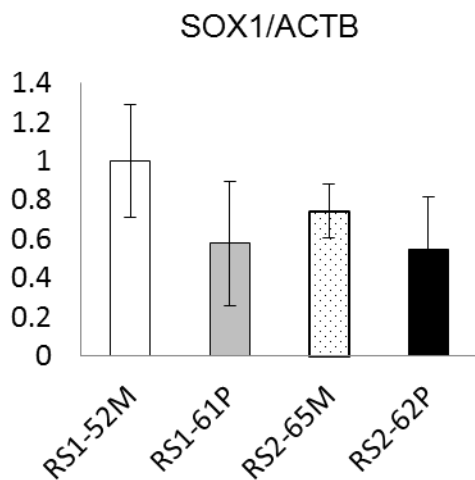

Supplement: Supplementary file 4 — Properties of neural stem cells derived from RTT-hiPSCs. (A) Diameter (n = 3) and (B) number of neurospheres (n = 16) derived from RTT-hiPSCs at 7 days in vitro. (C) The mRNA expression levels of the neural stem cell markers, NESTIN (NES) and SOX1, were quantified in RTT hiPSC-derived neurospheres by qPCR. Each value was normalized by comparison with ACTB expression and standardized by the value for the wild-type MECP2-expressing RS1-52 M clone, which was set to 1 (n = 4). Primer sets are listed in Additional file 5. Data were analyzed by Student’s t-test and Welch’s t-test (*p < 0.05). [file 13041_2015_121_MOESM4_ESM.pdf]
