# Supplementary material for: Differentiation of multipotent neural stem cells derived from Rett syndrome patients is biased toward the astrocytic lineage
Source: Mol Brain. 2015 May 27;8:31. doi: 10.1186/s13041-015-0121-2 (PMC4446051; doi:10.1186/s13041-015-0121-2)
Supplement: Supplementary file 5 — List of primers used in Additional file 3 and 4. [file 13041_2015_121_MOESM5_ESM.pdf]

**qPCR primers**
NESTIN-forward: TTCCCTCAGCTTTCAGGACCCCAA<br>*NESTIN*-reverse: AAGGCTGGCACAGGTGTCTCAA
*SOX*1-forward: GATCAGCAAGCGCCTGGGGG<br>*SOX1*-reverse: AGCAGCGTCTTGGTCTTGCGG
NR3C1-forward: GGACCACCTCCCAAACTCTG<br>*NR3C1*-reverse: GCTGTCCTTCCACTGCTCTT
